# Exploration of glycosyltransferases mutation status in cervical cancer reveals PARP14 as a potential prognostic marker

**DOI:** 10.1007/s10719-023-10134-7

**Published:** 2023-08-31

**Authors:** Hui Wang, Shen Luo, Xin Wu, Yuanyuan Ruan, Ling Qiu, Hao Feng, Shurong Zhu, Yanan You, Ming Li, Wenting Yang, Yanding Zhao, Xiang Tao, Hua Jiang

**Affiliations:** 1 Obstetrics & Gynecology Hospital of Fudan University, Shanghai, 200090 China; 2 Department of Biochemistry and Molecular Biology, School of Basic Medical Sciences, Fudan University, Shanghai, 200032 China; 3 Shanghai Genenexus healthcare technology company, Shanghai, 200433 China; 4 Department of Molecular and Systems Biology, The Geisel School of Medicine at Dartmouth, 03756 Lebanon, NH USA

**Keywords:** Glycosyltransferase (GT), Cervical Cancer, PARP14 (poly (ADP-ribose) polymerase member 14), Mutation

## Abstract

This study investigates the potential role of Glycosyltransferases (GTs) in the glycosylation process and their association with malignant tumors. Specifically, the study focuses on PARP14, a member of GTs, and its potential as a target for tumors in the diagnosis and treatment of cervical cancer. To gather data, the study used somatic mutation data, gene expression data and clinical information from TCGA-CESE dataset as well as tissue samples from cervical cancer patients. Further verification was conducted through RT-qPCR and immunohistochemistry staining on cervical cancer tissues to confirm the expression of PARP14. The study utilized Kaplan-Meier for survival analysis of cervical cancer patient and found significant mutational abnormalities in GTs. The high frequency mutated gene was identified as PARP14. RT-qPCR revealed significantly higher mRNA expression of PARP14 compared to precancerous tissue. Using IHC combined with Kaplan-Meier,patients in the PARP14 high expression group had a better prognosis than the low expression group. The study identified PARP14 as a frequently mutated gene in cervical cancer and proposed its potential role in diagnosis and treatment.

**Supplementary Information:**

The online version contains supplementary material available at 10.1007/s10719-023-10134-7.

## Introduction

Glycosyltransferases (GTs) are a large family of enzymes that play an important role in the process of glycosylation. [1]. GTs are present in the endoplasmic reticulum (ER) and Golgi apparatus. Currently, more than 200 genes encoding GTs have been identified and classified into different families such as fucosyltransferase, sialyltransferase and N-acetylglucosaminyltransferase [1, 2]. Previous studies have suggested that the abnormal expression of GT may be associated with various diseases, especially related with the proliferation, invasion, metastasis, epithelial-mesenchymal transition (EMT) and chemoresistance in malignant tumor [3–7]. Therefore, GTs have been suggested as crucial biomarkers for the timely detection and prediction of prognosis in various types of cancer. [4, 5, 7].

Cervical cancer is one of the malignant tumors of the reproductive system that seriously threatens women’s health. Its incidence and mortality rate are ranked in the 5th and 4th place in the female population, and the trend is still increasing [8, 9]. Recent studies have shown that the glycosylation varients are closely related to the proliferation, metastasis and drug resistance of cervical cancer [10, 11] and certain GTs have been associated with diagnosis and prognosis [12]. These fingdings suggest that GTs may play a significant role in cervical cancer and could potentially become new targets for diagnosis and treatment.

Poly (ADP-ribose) polymerase member 14 (PARP14), also known as ARTD8, BAL2, or CoaST6, belongs to the group of glycosyltransferases (GTs). In the past years, PARP14 was found to be associated with STAT6 activation, B-cell differentiation, and the JNK1/JNK2 signaling pathway. There has been growing interest in PARP14 as a potential target in allergic inflammation and tumors [13].PARP14’s role in inflammatory diseases including atopic dermatitis (AD), emphysema and asthma [14–16].PARP14 also plays an important role in tumors. PARP14 high expression in human primary pancreatic cancer (PCa) is associated with poor prognosis, and PARP14 promotes the proliferation and gemcitabine chemoresistance of pancreatic cancer cells through activation of NF-κ pathway [17]. In hepatocellular carcinoma (HCC) cells, PARP14 plays a role in promoting cell survival. One way it does this is by inhibiting the activity of JNK1, which is a pro-survival protein that functions as a serine/threonine kinase. This inhibition leads to a suppression of the nuclear function of downstream PKM2, which ultimately promotes the survival of tumor cells [18].

This paper analyzed 244 GTs that were previously identified [19] and examined the mutation and expression levels of GTs in both cervical cancer patients from the Cancer Genome Atlas (TCGA) database and clinical patients. The study identified several GTs that were linked to either positive or negative prognosis. In addition, the paper delves into the role of PARP14in the prognosis of cervical cancer.

## Materials and methods

*TCGA dataset*: We obtained a dataset from The Cancer Genome Atlas (TCGA, https://portal.gdc.cancer.gov) consisting of 178 tumor samples with somatic mutation data, 3 normal samples and 306 tumor samples with gene expression data and clinical information. The dataset was selected from TCGA-CESE. This study adheres to the publication guidelines provided by TCGA (http://cancergenome.nih.gov/publications/ publicationguidelines).

*Clinical tissue specimens*: The use of human tumor specimen was approved by the local Ethics Committee and informed consents were obtained from all patients. The clinical cohort contained data from 10 cervical cancer patients from the Obstetrics and Gynecology Hospital of Fudan University. We collected the clinical characteristic of the 10 patients of cervical cancer, with a mean age of: 51.8 years (Table 1). Of the 10 patients, 8 had squamous cell carcinoma and 2 had adenocarcinoma. Additionally, there were 5 patients in stage I, 2 in stage II and 3 in stage III (Table 1). We performed whole genome sequencing (WGS) on the samples to obtain data related to their somatic mutations. Additionally, we established a cohort of 35 cervical cancer samples and a cohort of 4 cervical cancer tissues which were matched with para-cancer tissues using immunohistochemistry staining and RT-qPCR, respectively. All clinical specimens were untreated with preoperative chemotherapy or radiotherapy.

**Table 1:** Patient Characteristics Associated with Cervical Cancer of Clinical Cohort for WGS

Characteristic	No. (%)
Age (years), mean(range)	51.80(35–75)
≥ 70	1(10.00)
50–69	4(40.00)
35–49	5(50.00)
＜35	0(0.00)
Body mass index(kg/m2)	23.85(18.69–26.44)
BMI < 18.5	0(0.00)
18.5 ≤ BMI＜24	4(40.00)
BMI ≥ 24	6(60.00)
ECOG performance status score	
0	9(90.00)
1	1(10.00)
≥ 2	0(0.00)
Menstruation	
Menopause	5(50.00)
Pre-menopause	5(50.00)
Marital status	
Single	0(0.00)
Married or Divorced	10(100.00)
Histologic subtypes	
Squamous carcinoma	8(80.00)
Adenocarcinoma	2(20.00)
FIGO stage	
I	5(50.00)
II	2(20.00)
III	3(30.00)
IV	0(0.00)

*Quantitative Real-Time PCR Analysis*: The mRNA of cervical cancer tissue was isolated and reverse transcripted to cDNAs by the MolPure® Cell/Tissue Total RNA Kit (19221ES50, Yeasen, Shanghai, China) and Hifair® II 1st Strand cDNA Synthesis Kit (gDNA digester plus) (11123ES10, Yeasen, Shanghai, China). Then the cDNAs was used for quantitative PCR analysis using Hieff® qPCR SYBR Green Master Mix(Low Rox Plus) (11202ES03, Yeasen, Shanghai, China).The RNA concentration and quality were determined by NanoDrop 2000 (Applied Biosystems, USA)and the RT-qPCR analysis was performed on QuantStudio5 Real-Time PCR (Applied Biosystems, USA). The PCR primers were as follows: GAPDH forward, 5’-GGAGCGAGATCCCTCCAAAAT-3’ and reverse, 5’-GGCTGTTGTCATACTTCTCATGG-3’; PRAP14 forward, 5’ -TGTTAGTGGAGAACATAAGTGGC-3’ and reverse, 5’ -TGAATGGTGCTTGGTACAATCAT-3’. GAPDH was used as the internal control. Data were analyzed using the 2-ΔΔCt method.

*Immunohistochemistry (IHC) Staining*: The samples from the tissue were embedded in paraffin. Tissue sections (3 μm) were prepared for IHC analyses of PARP14. In brief, the sections were de-paraffinized and, dehydrated, and were then subjected to antigen retrieval. Endogenous peroxidase was eliminated by the use of 3% H2O2.The slides were then incubated with anti-PARP14(1:100, Rabbit, Affinity, DF14173) overnight at 4℃. The samples were incubated with a second antibody (Mouse anti-rabbit IgG, Recordbio, RC0080RM) at room temperature, developed with DAB, counterstained with hematoxylin for 3 min, and photographed under a microscope. All images were acquired and processed in TIFF format, and the integrated option densities (IODs) of in each image were counted and measured using ImageJ (1.53t, Java 1.8.0_322) and IHC-Toolbox plugin [20].

### Gene set enrichment analysis (GSEA):

Gene set enrichment analysis (GSEA) is a commenly used computational method for analyzing genome-wide expression matrices [21]. In this study, we utilized GSEA to assess related pathways and molecular mechanisms in cervical cancer patients by setting GTs gene mutation levels as population phenotypes. The nominal P-value and normalized enrichment score (NES) were used to sort the pathways enriched in each phenotype. Gene sets with a nominal P value of less than 0.05 were considered statistically significant.

*Statistics Analysis*: The R software (4.0.2, http://www.R-project.org, RRID:SCR_001905) was used to derive all statistical analyses. Somatic mutation data for 178cervical cancer samples from TCGA-CESE cohort and 10 samples from the clinical cohort were extracted by “Maftools” plugin. Overall survival (OS) was predicted using Kaplan-Meier survival plots. GraphPad Prism V8.0.1 (GraphPad Software, Inc., La Jolla, CA, USA, RRID:SCR_002865) software was used to determine statistical differences by using the Welch’s t test or one-way analysis of variance (ANOVA). p < 0.05 was considered to be statistically significant.

## Results

### Glycosyltransferase (GT) genes Associated with Mutation in TCGA cohorts

**Fig. 1:**
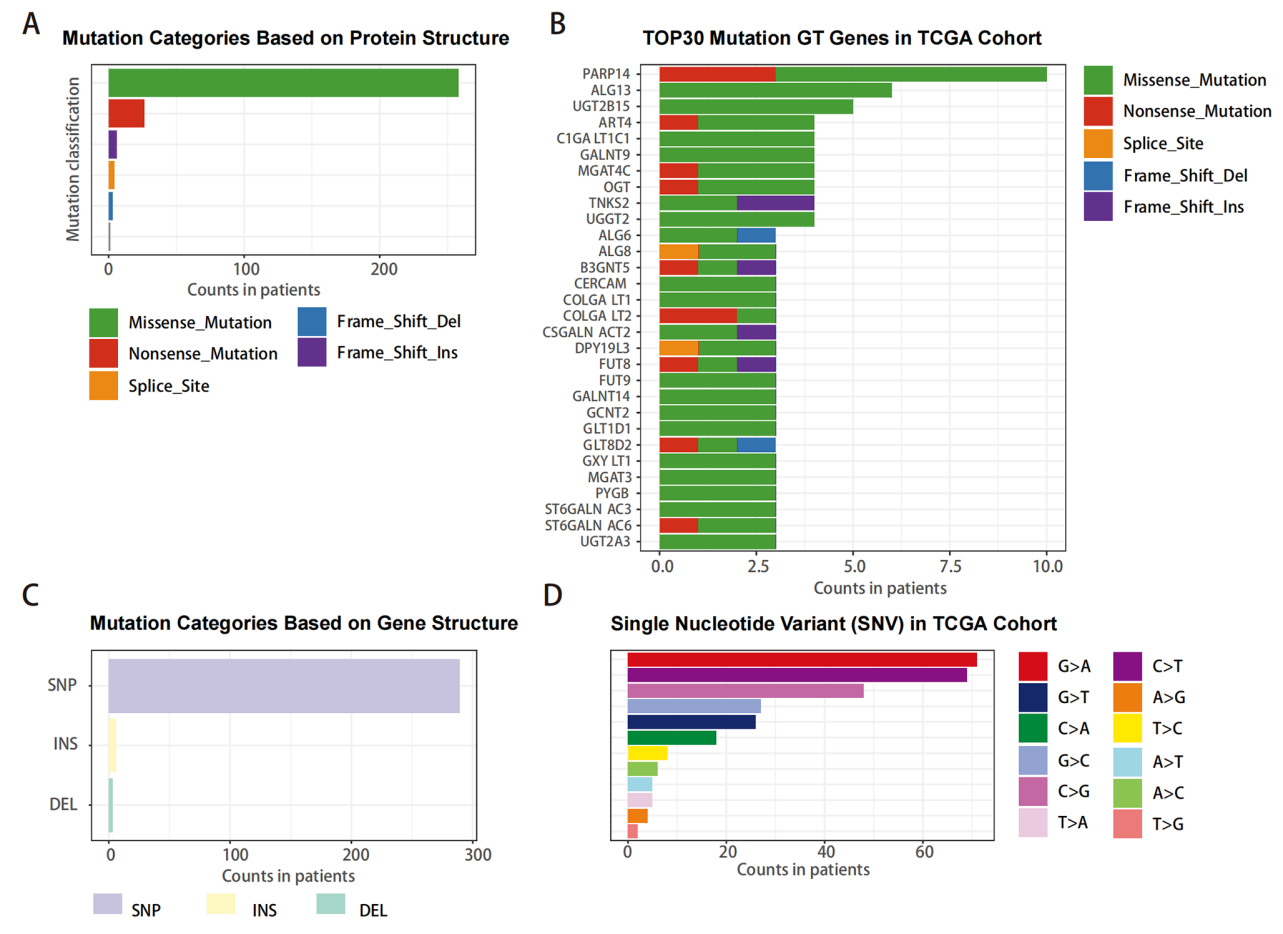
Summary of the GT mutation information from TCGA **(A)** Mutation types were classified by protein structure categories, with missense mutations accounting for the largest proportion. **(B)** The top 30 mutated GT genes in cervical cancer. **(C, D)** Mutation types were classified by gene structure categories, SNP occurring more frequency than insertion or deletion, and G > A being the most common mutation type in SNV

We analyzed the association between the mutation of 244 GTs and cervical cancer in TCGA cohorts. Somatic mutation profiles from 178 cervical cancer patients were downloaded and analyzed using “Maftools” plugin. The mutations were classified into different categories with missense mutations accounting for the largest proportion based on protein structure (Fig. 1A). Here we presented the top 30 mutated genes in cervical cancer, ranked by percentage, the top 10 including PARP14, ALG13, UGT2B15, ART4, C1GALNT1C1, GALNT9, MGAT4C, OGT, TNKS2, UGGT2 (Fig. 1B). In addition, single nucleotide polymorphisms (SNPs) occurred more frequently than insertions or deletions (Fig. 1C), with G > A being the most common single nucleotide variant (SNV) in cervical cancer (Fig. 1D) according to gene structure classification.

### Enrichment analysis of GTs based on TCGA cohort

**Fig. 2:**
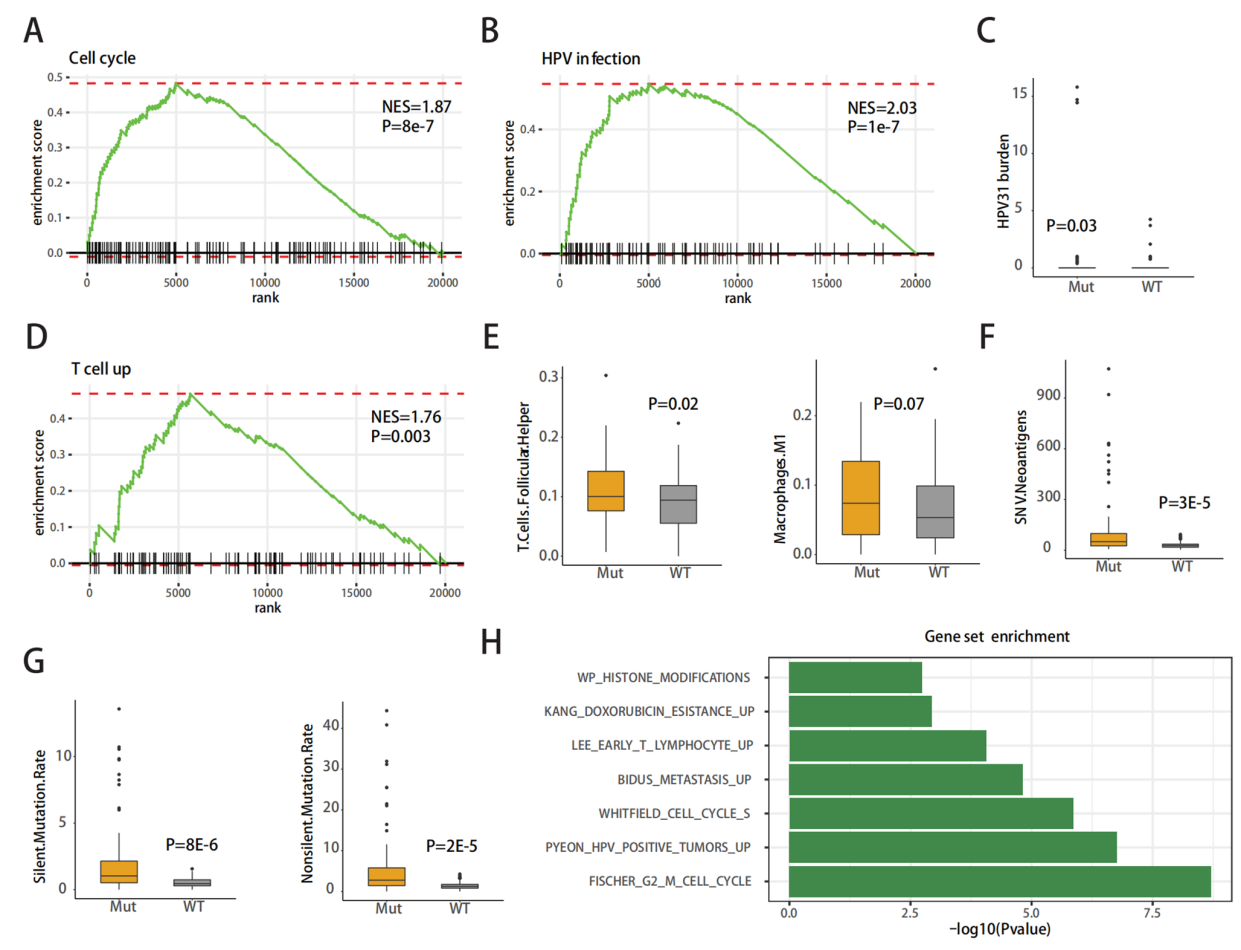
Enrichment analysis of GTs in TCGA cohort **(A)** GSEA Enrichment plot of Cell cycle in mutation group. **(B)** GSEA Enrichment plot of HPV infection in mutation group. **(C)** Comparison of HPV31 burden between GTs mutant-type and wild-type cervical cancer patients in TCGA cohorts. **(D)** GSEA Enrichment plot of T cell in mutation group. **(E)** Comparison of T-helper cells follicular and M1 macrophages between GTs mutant-type and wild-type cervical cancer patients in TCGA cohorts. **(F)** SNV-induced neoantigen was significantly higher in the mutant group, which may be the reason for the increase in T cells. **(G)** Both silent and non-silent mutations were significantly increased in mutant-type compared to wild-type. **(H)** Name of the gene set with the most significant enrichment differences in the GEEA gene set

To investigate the potential mechanism of GT effect on cervical cancer, we utilized Gene Set Enrichment Analysis (GSEA) to examine the pathways and biological processes associated with GTs. We divided the 178 cervical cancer samples from the TCGA database into mutation and non-mutation groups based on the mutation status of 244 GTs. Out of these patients, 104 were classified as the GTs mutant-type group while 90 were classified as the GTs wild-type group. As shown, we obtained significant enrichment in cell cycle (NES = 1.87, P = 8e-7), HPV infection (NES = 2.03, P = 1e-7) and T cell (NES = 1.76, P = 0.03) pathways (Fig. 2A,B,D). In HPV infection analysis, we further found that HPV31 infection was higher in mutant-type than wild-type (Fig. 2C). In our investigation of T cell-related pathways, we observed an increase in T.helper cell and M1 macrophage in mutant-type(Fig. 2E), and further analysis revealed that the increase in SNV neoantigen may be the reason for the high T cell content(Fig. 2F). In addition, the ratio of silent and non-silent mutations was significantly elevated in the mutant-type (Fig. 2G). Finally, we identified the gene sets with the most significant differences in the enrichment of GSEA genes, including histone modifications, doxorubicin resistance, early T lymphocyte, metastasis, cell cycle, HPV positive tumors and G2M cell cycle(Fig. 2H).

### Glycosyltransferase (GT) genes Associated with mutation in clinical cohorts

**Fig. 3:**
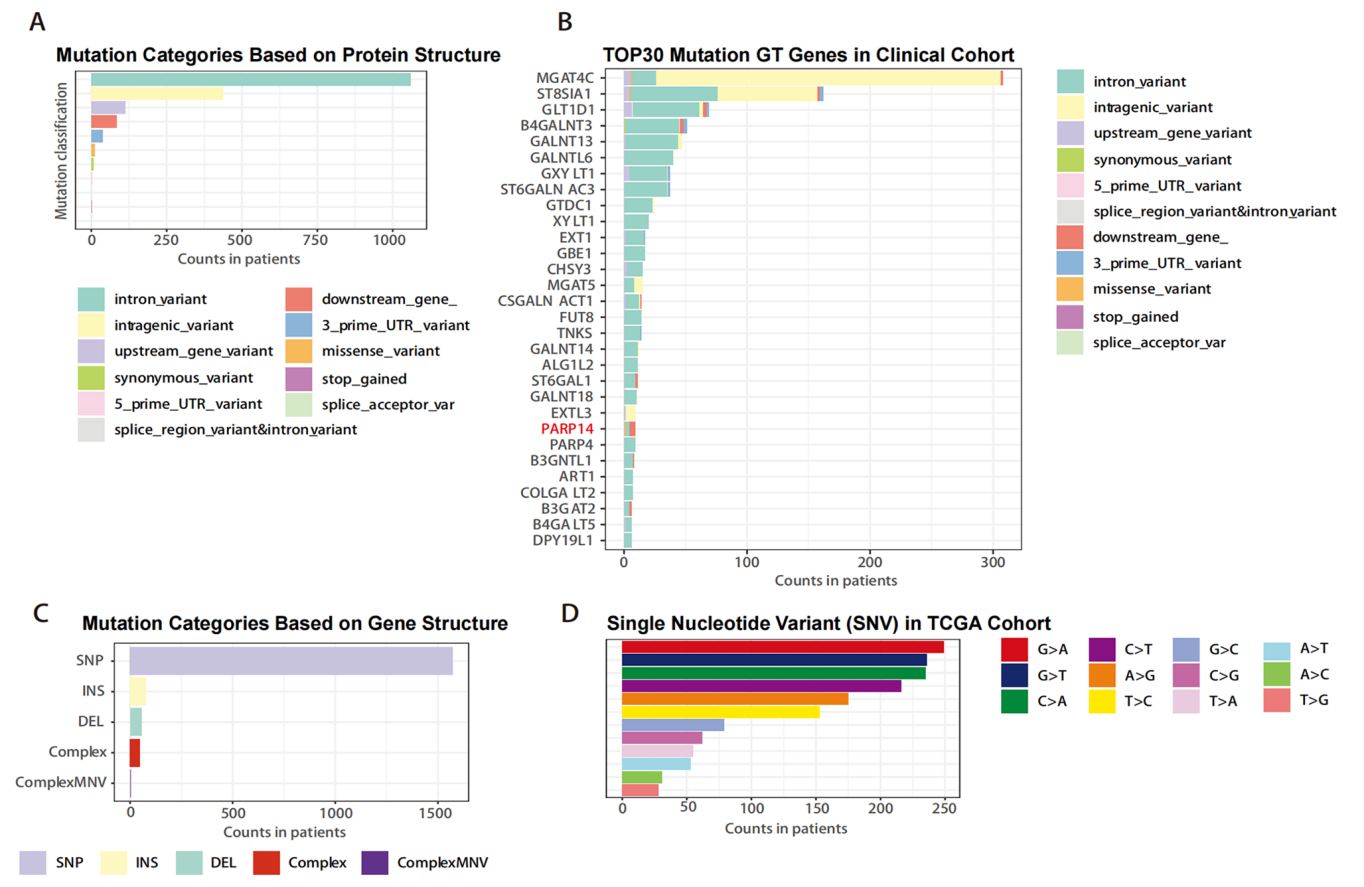
Somatic mutation patterns of GTs identified by whole-genome sequencing (WGS) in 10 patients of cervical cancer. **(A)** Mutation types were classified by protein structure categories, with intron variant accounting for the largest proportion. **(B)** The top 30 mutated GT genes in cervical cancer. **(C, D)** Mutation types were classified by gene structure categories, SNP occurring more frequency than insertion or deletion, and G > A being the most common mutation type in SNV

We conducted whole genome sequencing of 10 cervical cancer tissues, and obtained somatic mutation data for analysis. These mutations were classified into different categories, with the largest proportion of intron variants based on protein structure (Fig. 3A). We identified the top 30 mutated genes, sorted by percentage, and the top 10 included MGAT4C, ST8SIA1, GLT1D1, B4GALNT3, GALNT13, GALNTL6, GXYLT1, ST6GALNAC3, GTDC1, XYLT (Fig. 3B). In addition, SNP occurred more frequently than insertions or deletions (Fig. 3C), with G > A being the most common SNV in cervical cancer (Fig. 3D). To compare with the mutations in the TCGA cohort, we screened for GT genes with missense mutations. A total of 12 GT genes in the clinical cohort had missense mutations, including PARP14 (Table 2).

**Table 2:** Glycosyltransferase Genes with Missense Mutations in Clinical Cohort For WGS.

Glycosyltransferase (GT) Genes
A4GNT
ALG10
ALG10B
B3GALNT2
DPY19L2
GALNT6
LFNG
MGAT4C
PARP12
PARP14
ST6GAL2
ST8SIA1

### Somatic mutation of GTs from patients with cervical cancer in TCGA and clinical cohort

**Fig. 4:**
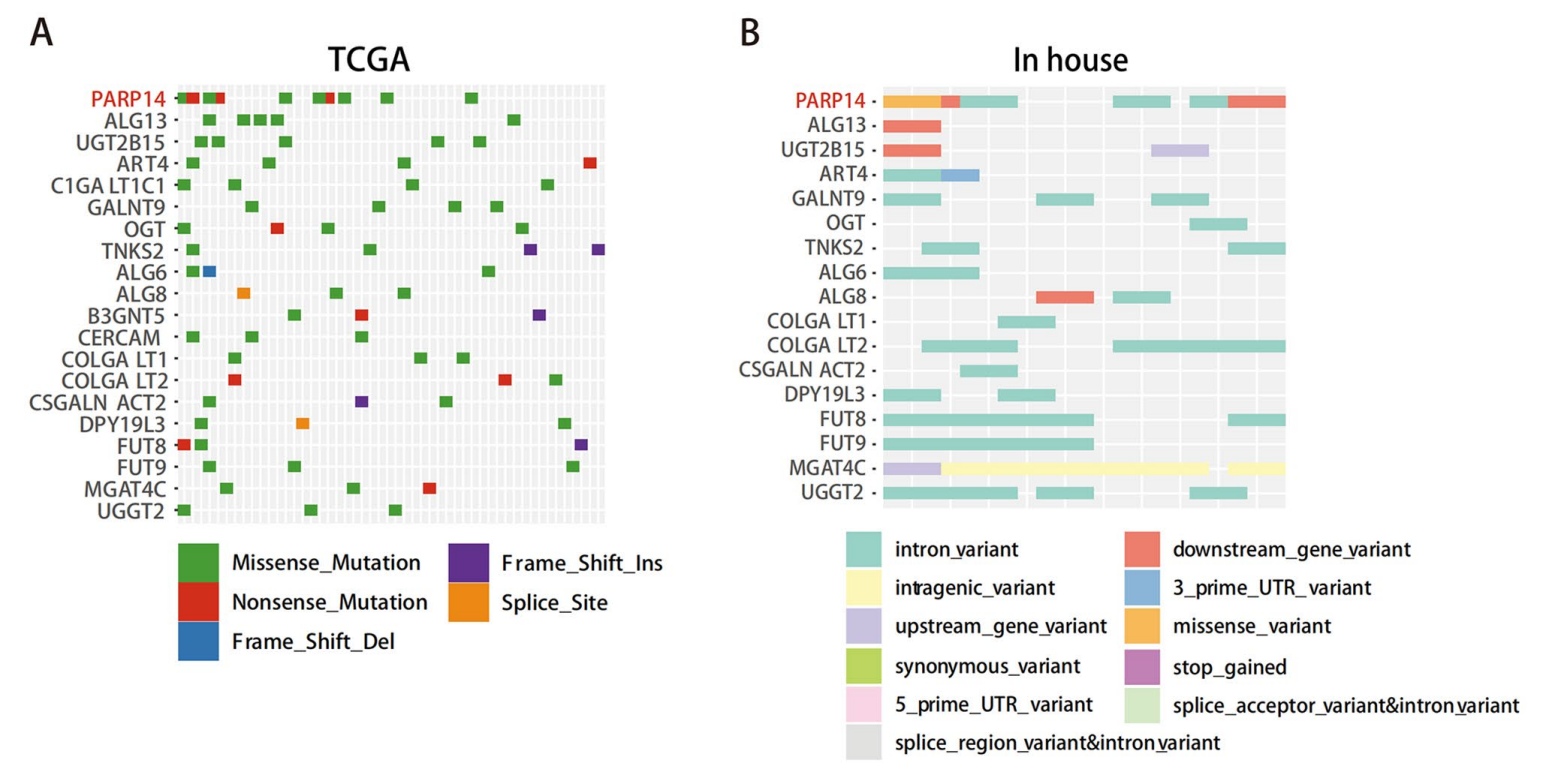
Mutation and prognosis analysis of glycosyltransferase PARP14 in cervical cancer. **(A, B)** The most recurrently mutated GTs genes and the somatic mutation profiles identified from TCGA and tissues of cervical cancer patients. Cases are shown in columns, genes in rows

Furthermore, we summarized the mutation categories of the most frequently mutated GTs in TCGA datasets and tissues (Fig. 4A, B). Notably, PARP14 had a high frequency of missense mutations in both cohorts, despite not having a higher mutation frequency in the tissue cohort. This arouse our interest in investigating the role of PARP14 in cervical cancer, leading to the following related studies.

### PARP14 expression in cervical cancer tissues and correlation analysis

**Fig. 5:**
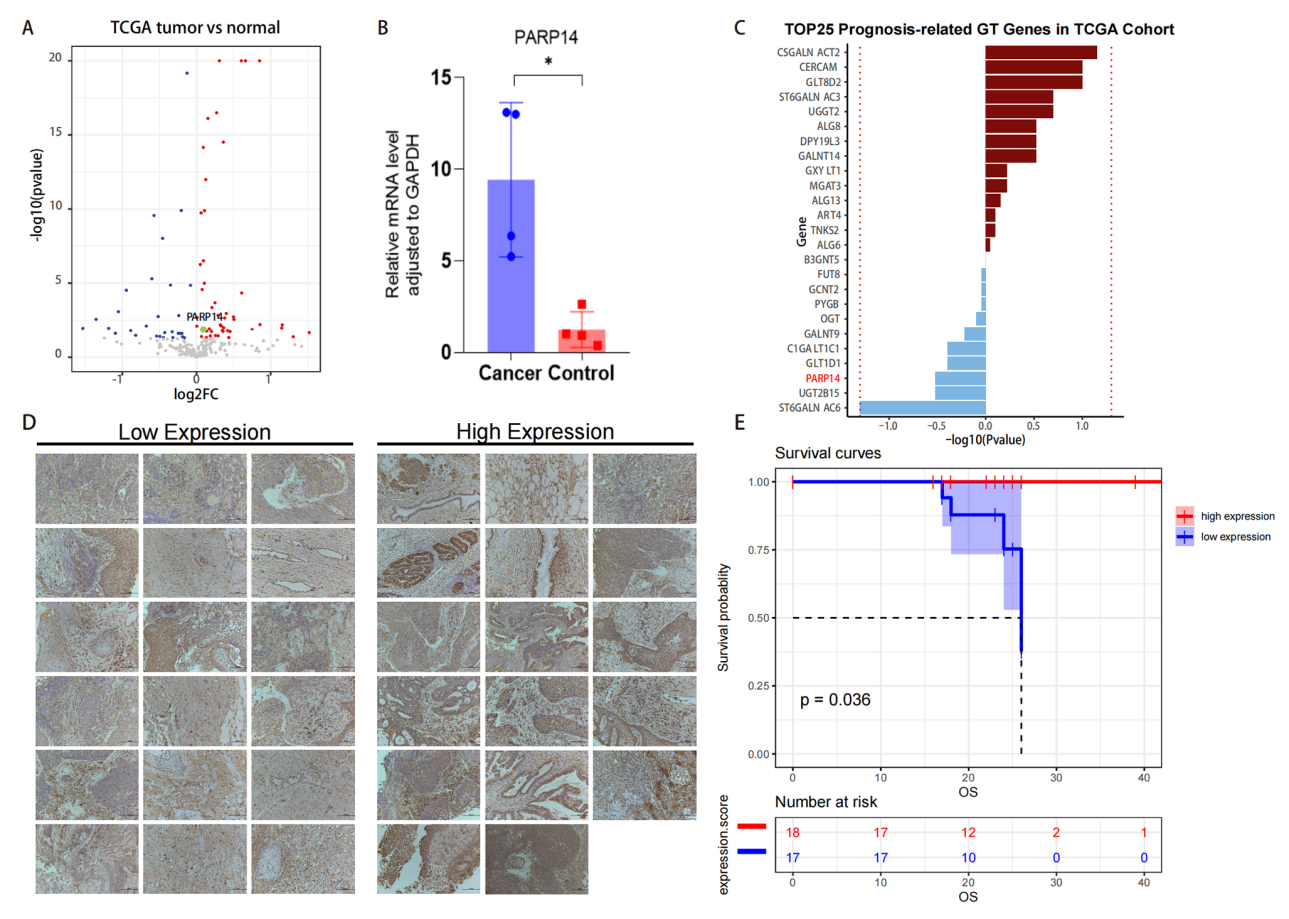
PARP14 expression in cervical cancer tissues and correlation analysis. **(A)** Volcano plot of mRNA expression of GTs in tumor and normal cervical tissues from TCGA dataset. In the volcano map, red: genes up-regulated in tumor group; blue: genes down-regulated in tumor groups; gray: no differentially expressed genes in the tumor group; green: PARP14. **(B)** PARP14 expression in tumor and normal cervical tissues. **(C)** GT genes which had the greatest protective or hazardous effect on the prognosis of cervical cancer from TCGA cohort. **(D)** Immunohistochemical staining of PARP14 protein was performed on 35 cervical cancer tissues, which were divided into low expression group (N = 18) and high expression group (N = 17) according to expression level. **(E)** Kaplan-Meier survival curves for overall survival of cervical cancer patients from two groups

According to TCGA datasets, there was an up-regulation of PARP14 in cervical cancer when compared to normal tissues (Fig. 5A). And it was also validated that the mRNA expression of PARP14 in tumor tissues was significantly increased when compared to the matched para-cancer tissues (Fig. 5B). To investigate the association between glycosyltransferase mutations and cervical cancer prognosis, we further conducted a survival analysis of 244 GTs in the TCGA cohort. Our analysis revealed that the majority of the 244 GTs had either a protective or hazardous effect on the prognosis of cervical cancer (Online Resource 1, Online Resource 2). We listed the 25 GT genes in which mutations had the greatest protective or hazardous effect on the prognosis of cervical cancer, with mutations in the PARP14 gene exhibiting a protective effect against cervical cancer (Fig. 5C). At the same time, we performed immunohistochemical staining of cancer tissues from a clinical cohort of 35 cervical cancer patients to analyze the qualitative and quantitative expression levels of PARP14. Clinical characteristics and prognostic data were collected. The median follow-up time was 23 months. We divided them into PARP14 high expression group (N = 17) and PARP14 low expression group (N = 18) (Fig. 5D) based on quantitative immunohistochemical analysis. The study utilized the Kaplan-Meier method to assess the prognosis of two groups, and the results were statistically different (p = 0.036), with patients in the high PARP14 expression group having a better prognosis compared to those in the low PARP14 expression group (Fig. 5E). In addition, we also conducted a differential analysis based on various factors such as pathological type, clinicopathological stage, menopause and body mass index (BMI). However, there was no significant difference in PARP14 expression among any of these factors(Table 3).

**Table 3:** Patient Characteristics Associated With Cervical Cancer Of Clinical Cohort For IHC.

Characteristic	Total (n = 35)	High expression (n = 17 )	Low expression (n = 18 )
	No. (%)	No. (%)	No. (%)
Age (years), mean(range)	50.94(31–72)	51.41(31–72)	50.50(36–72)
≥ 70	2(5.71)	1(5.88)	1(5.56)
50–69	18(51.43)	9(52.94)	9(50.00)
35–49	13(37.14)	5(29.41)	8(44.44)
＜35	2(5.71)	2(11.76)	0(0.00)
Body mass index(kg/m2)	24.19 (20.51–32.47)	24.06 (20.51–32.47)	24.33 (19.63–29.90)
BMI < 18.5	0(0.00)	0(0.00)	0(0.00)
18.5 ≤ BMI＜24	22(62.86)	11(64.71)	11(61.11)
BMI ≥ 24	13(37.14)	6(35.29)	7(38.89)
ECOG performance status score			
0	34(97.14)	16(94.12)	18(100.00)
1	1(2.86)	1(5.88)	0(0.00)
≥ 2	0(0.00)	0(0.00)	0(0.00)
Menstruation			
Menopause	14(40.00)	6(35.29)	8(44.44)
Pre-menopause	21(6.000)	11(64.71)	10(55.56)
Marital status			
Single	2(5.71)	1(5.88)	1(5.56)
Married or divorced	33(94.29)	16(94.12)	17(94.44)
Histologic subtypes			
Squamous carcinoma	28(80.00)	13(76.47)	15(83.33)
Adenocarcinoma	5(14.29)	2(11.76)	3(16.66)
Adenosquamous carcinoma	2(5.71)	2(11.76)	0(0.00)
FIGO stage			
I	12(34.29)	6(35.29)	6(33.33)
II	10(28.57)	3(17.65)	7(38.89)
III	13(37.14)	8(47.06)	3(16.66)
IV	0(0.00)	0(0.00)	0(0.00)
Follow-up (month), median (range)	23(0–42)	23(17–26)	23(0–42)

## Discussion

Glycosyltransferases are a large group of enzymes which play a crucial role in glycosylation. Over 200 GTs have been identified and their abnormal expression has been closely related to tumors. In colorectal cancer, the expression of GTs has been found to contribute significantly to tumor cell proliferation, survival, stem-like cell properties induction, epithelial-mesenchymal transition (EMT), metastasis and resistance to chemotherapy and radiotherapy [4]. Jahanshah Ashkani et al. analyzed that the expression of 210 GT genes from 1893 cancer patient samples in The Cancer Genome Atlas (TCGA) microarray data. Their findings showed that these genes were effective in classifying six types of cancers, including breast, ovarian, glioblastoma, kidney, colon and lung. Furthermore, these GT genes were able to classify subgroups of breast cancer [22]. Yousra Mohamed Abd-El-Halim et al. proposed a gene signature funded based on 19 GT genes that can effectively stratify the prognosis of patients with pancreatic ductal adenocarcinoma (PDAC) [23]. These studies indicates that the expression of GTs has an impact on the fate of tumors.

Our study summarized the mutation pattern of GTs in cervical cancer tissues based on TCGA database and whole genome sequencing (WGS). According to the analysis of TCGA database, it revealed that missense mutations were the most common protein structure mutation types in GTs, with the top 30 mutation frequencies all having missense mutations. Meanwhile, the number of SNPs was also more than other gene structure mutation types. Missense variants were the most common consequence of SNPs in humans [24]. We concerned that the effect of missense mutations on glycosyltransferase activity was arguably more difficult to judge than in the case of mutations with other consequences (such as total gene deletions or point nonsense mutation) [25]. Among the top 30 GTs in terms of mutation frequency, a de-novo missense mutation in C1GALT1C1: c.266 C > T, p. (T89I) caused a novel X-linked form of atypical hemolytic-uremic syndrome (aHUS) [26]. Another study by Marilena De Mariano et al. revealed that a GALNT14 mutation (c.802 C > T) had been associated to the development of neuroblastoma (NB) and GALNT14 might act as a novel gene potentially involved in NB predisposition [27].

Recent studies confirmed the correlation between GTs and cervical cancer. UDP-glucose ceramide glycosyltransferase (UGCG) contributed to proliferation and glycolysis of cervical cancer cells by regulating the PI3K/AKT pathway [10]. Additionally, Lixia Zhou et al. demonstrated an increase in the mRNA expression and protein level of GALNT2 in cervical high-grade intraepithelial neoplasia and tumor tissues compared to normal cervix tissues. GALNT2 was associated with worse overall survival and could be utilized as a prognostic biomarker for cervical cancer [12]. The findings of the current study confirmed that there was a correlation between the expression of GTs and the prognosis of cervical cancer. Out of the 244 GTs analyzed, almost all of them were found to have either a protective or detrimental effect on the prognosis of cervical cancer.

PARP14, a member of the glycosyltransferase family, has been shown to be closely associated with the development of a number of tumors, particularly in pancreatic cancer, hepatocellular carcinoma, and hematologic malignancy [17, 18, 28–30]. In diffuse large B-cell lymphoma (DLBCL), PARP14 regulated the IL-4-STAT6 signaling pathway, enhanced the expression of several downstream genes, and enabled DLBCL tumor cell survival [31]. In this study, we found that PARP14 showed a trend of high expression in cervical cancer tissues compared to para cancerous tissues, and the high expression of PARP14 was closely related to the prognosis of patients, suggesting that patients with high PARP14 expression have a better prognosis than those with low expression. Although our case sample was limited and our study was restricted to a descriptive study, the result still led us to focus on the role of PARP14 in cervical cancer. GSEA enrichment analysis identified cell cycle, HPV infection, and T cell-associated immunity as the probable mechanistic pathways. It had been demonstrated that cyclin D1 was over-expressed in many tumors. PARP14 deletion led to a decrease of cyclin D1, resulting in G1 cell-cycle arrest and reduced proliferation [32]. This confirmed the involvement of PARP14 in regulating cell cycle progression. However, the role of this mechanism in cervical cancer was unclear. Among many allergic diseases, PARP14 interacted with STAT6 to enhance IL-4-induced gene expression in T cells, thereby promoting Th2 differentiation [33, 34]. Research on the mechanism of PARP14 and HPV infection has not known yet. Regrettably, our study has not yet established a correlation between mutation and expression of PARP14 in cervical cancer. This would hold significant importance for future studies. The potential mechanism of PARP14 in cervical cancer will be explored in our further studies.

## Conclusions

In this study, we analyzed the high-frequency mutated GT gene PARP14 from both TCGA database and clinical case samples. We found that PARP14 was significantly overexpressed at both the molecular and protein levels in cervical cancer tissues, and that higher expression of PARP14 caused better prognosis. Our findings suggested a potential role of PARP14 in the diagnosis and treatment of cervical cancer.

### Electronic supplementary material

Below is the link to the electronic supplementary material.

Supplementary Material 1

Supplementary Material 2

Supplementary Material 3

## Data Availability

The datasets generated during and/or analyzed during the current study are available from the corresponding author on reasonable request.
