# Supplementary material for: Exploration of glycosyltransferases mutation status in cervical cancer reveals PARP14 as a potential prognostic marker
Source: Glycoconj J. 2023 Aug 31;40(5):513–22. doi: 10.1007/s10719-023-10134-7 (PMC10638145; doi:10.1007/s10719-023-10134-7)
Supplement: Supplementary file 1 — Supplementary Material 1 [file 10719_2023_10134_MOESM1_ESM.pdf]

Title: Exploration of glycosyltransferases mutation status in cervical cancer reveals PARP14 as a potential prognostic marker

Hui Wang [1 †], Shen Luo [1 †], Xin Wu [1],Yuanyuan Ruan [2], Ling Qiu [1], Hao Feng [1], Shurong Zhu [1], Yanan You [1], Ming Li [1], Wenting Yang [3], Yanding Zhao [4], Xiang Tao [1], Hua Jiang [1,*]

1 Obstetrics & Gynecology Hospital of Fudan University, Shanghai 200090, China;

2 Department of Biochemistry and Molecular Biology, School of Basic Medical Sciences, Fudan University, Shanghai 200032, China

3 Shanghai Genenexus healthcare technology company, Shanghai 200433, China;

4 Department of Molecular and Systems Biology, The Geisel School of Medicine at Dartmouth, Lebanon, NH 03756；

* Correspondence: jianghua@fudan.edu.cn(H.J.).

† These authors contributed equally to this work.

Journal Name: Glycoconjugate Journal

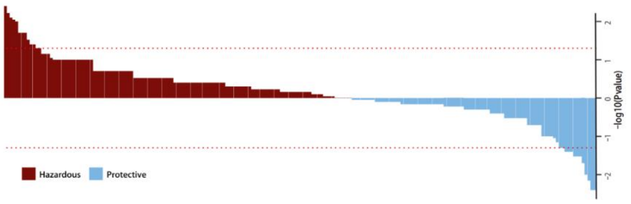

Figure S1: Summary of the correlation between GTs and the prognosis of cervical cancer based on TCGA cohort.
