# Supplementary material for: Exploration of glycosyltransferases mutation status in cervical cancer reveals PARP14 as a potential prognostic marker
Source: Glycoconj J. 2023 Aug 31;40(5):513–22. doi: 10.1007/s10719-023-10134-7 (PMC10638145; doi:10.1007/s10719-023-10134-7)
Supplement: Supplementary file 2 — Supplementary Material 2 [file 10719_2023_10134_MOESM2_ESM.pdf]

Title: Exploration of glycosyltransferases mutation status in cervical cancer reveals PARP14 as a potential prognostic marker

Hui Wang [1 †], Shen Luo [1 †], Xin Wu [1], Yuanyuan Ruan [2], Ling Qiu [1], Hao Feng [1], Shurong Zhu [1], Yanan You [1], Ming Li [1], Wenting Yang [3], Yanding Zhao [4], Xiang Tao [1], Hua Jiang [1,*]

1 Obstetrics & Gynecology Hospital of Fudan University, Shanghai 200090, China;

2 Department of Biochemistry and Molecular Biology, School of Basic Medical Sciences, Fudan University, Shanghai 200032, China

3 Shanghai Genenexus healthcare technology company, Shanghai 200433, China;

4 Department of Molecular and Systems Biology, The Geisel School of Medicine at Dartmouth, Lebanon, NH 03756；

* Correspondence: jianghua@fudan.edu.cn(H.J.).

† These authors contributed equally to this work.

Journal Name: Glycoconjugate Journal

Table S1: Prognosis-related Glycosyltransferase(GT) Genes in TCGA Cohorts.

Glycosyltransferase(GT) Genes	Hazard Ratio	P value
FUT7	0.35	0.004
GALNT3	3	0.004
PARP6	0.32	0.004
DPY19L4	2.8	0.006
A4GALT	0.36	0.007
FUT11	2.7	0.008
UGGT1	2.7	0.009
GYG2	2.5	0.01
RFNG	0.36	0.01
C1GALT1	2.4	0.02
KDELC1	2.4	0.02
MGAT5	2.5	0.02
PIGZ	0.42	0.02
B3GAT2	2.4	0.03
B3GNTL1	0.45	0.03
HPRT1	0.44	0.03
PIGQ	0.43	0.03
ALG1L	0.48	0.04
B3GAT1	0.48	0.04

CHSY3	2.2	0.04
POFUT2	2.1	0.04
ST3GAL3	0.47	0.04
ALG11	2.1	0.05
B3GALT4	0.49	0.05
GALNTL6	2.1	0.05
ST6GALNAC6	0.49	0.05
CSGALNACT2	2	0.07
FUT5	0.52	0.07
HAS2	2	0.07
MGAT2	2	0.07
B4GALT2	0.54	0.09
GALNT5	1.9	0.09
ALG1	1.8	0.1
B3GNT2	1.8	0.1
B4GALT1	1.7	0.1
CERCAM	1.7	0.1
GALNT10	1.8	0.1
GALNT7	1.8	0.1
GBE1	1.7	0.1
GLT8D2	1.8	0.1
GYG1	0.55	0.1
PPAT	1.8	0.1
ST3GAL1	1.7	0.1
ST6GALNAC2	0.59	0.1
ST8SIA1	0.55	0.1
ST8SIA2	1.7	0.1
STT3B	1.7	0.1
UGCG	1.8	0.1
UGT3A2	1.7	0.1
UMPS	0.59	0.1
B3GALT5	1.6	0.2
B3GNT3	1.6	0.2
B3GNT9	1.7	0.2
CHPF	1.6	0.2
CHSY1	0.64	0.2
EXT1	1.7	0.2

GCNT3	1.7	0.2
GXYLT2	1.5	0.2
MGAT4A	1.5	0.2
PARP12	0.62	0.2
PARP15	0.65	0.2
PARP4	1.6	0.2
PIGH	1.6	0.2
POFUT1	1.6	0.2
ST3GAL5	0.63	0.2
ST6GALNAC3	1.6	0.2
UGGT2	1.6	0.2
UGT1A6	0.65	0.2
UGT8	1.6	0.2
ALG8	1.4	0.3
ALG9	1.4	0.3
APRT	0.7	0.3
ART3	0.71	0.3
B3GNT6	1.5	0.3
B4GALNT2	1.4	0.3
DPY19L1	1.5	0.3
DPY19L3	1.5	0.3
FUT3	1.4	0.3
GALNT14	1.4	0.3
GCNT4	1.5	0.3
GTDC1	1.5	0.3
PARP14	0.72	0.3
PIGC	1.5	0.3
QTRT1	0.68	0.3
ST6GAL2	1.5	0.3
ST8SIA4	0.7	0.3
ST8SIA6	1.4	0.3
TMEM5	1.4	0.3
UGT1A9	0.68	0.3
UGT2A1	0.68	0.3
UGT2B15	0.67	0.3
ALG12	1.3	0.4
ALG14	1.4	0.4

ALG2	1.4	0.4
B3GAT3	0.75	0.4
B4GALNT4	1.3	0.4
B4GALT4	1.4	0.4
C1GALT1C1	0.75	0.4
CSGALNACT1	1.3	0.4
EXTL1	1.3	0.4
EXTL2	1.3	0.4
EXTL3	0.74	0.4
GALNT11	1.4	0.4
GALNT13	1.4	0.4
GALNT1	1.3	0.4
GALNT4	1.3	0.4
GCNT1	1.3	0.4
GLT1D1	0.75	0.4
MTAP	1.4	0.4
PARP8	1.3	0.4
PIGB	1.3	0.4
PIGM	1.4	0.4
POMT2	1.4	0.4
ST3GAL6	0.73	0.4
AGL	1.3	0.5
ALG10B	1.3	0.5
B3GALNT1	0.81	0.5
B3GNT4	1.2	0.5
B4GALT3	1.3	0.5
B4GALT6	1.3	0.5
CD38	0.8	0.5
EXT2	0.79	0.5
FUT1	0.81	0.5
HAS1	1.3	0.5
LFNG	0.8	0.5
MGAT4B	1.3	0.5
MGAT5B	0.79	0.5
PIGV	1.2	0.5
PYGM	0.77	0.5
SIRT4	0.8	0.5

ST6GALNAC5	1.3	0.5
ST8SIA5	0.78	0.5
ALG1L2	0.82	0.6
ART5	1.2	0.6
B4GALNT1	0.84	0.6
CHPF2	0.81	0.6
DPY19L2	1.2	0.6
FUT2	0.84	0.6
FUT6	0.82	0.6
GALNT9	0.82	0.6
GXYLT1	1.2	0.6
GYS2	1.2	0.6
MGAT3	1.2	0.6
PARP10	0.85	0.6
PIGP	1.2	0.6
ST3GAL4	1.2	0.6
STT3A	1.2	0.6
TNKS	1.2	0.6
XYLT1	1.2	0.6
ABO	1.1	0.7
ALG13	1.1	0.7
ALG3	0.89	0.7
ALG5	1.2	0.7
B3GALNT2	1.1	0.7
B3GALT2	0.89	0.7
B3GALT6	0.86	0.7
B3GNT7	1.2	0.7
B3GNT8	0.86	0.7
B4GALT7	0.88	0.7
DPM1	0.89	0.7
FUT10	1.2	0.7
GALNT12	1.1	0.7
GALNT2	1.2	0.7
GALNT6	1.2	0.7
GLT8D1	0.86	0.7
GYS1	0.88	0.7
MFNG	0.88	0.7

MGAT1	0.86	0.7
NAMPT	1.2	0.7
PARP16	0.89	0.7
PIGA	0.85	0.7
ST3GAL2	0.86	0.7
ST6GALNAC1	1.1	0.7
UPP1	0.86	0.7
XYLT2	0.86	0.7
ART4	1.1	0.8
GBGT1	1.1	0.8
GCNT7	0.91	0.8
HAS3	0.91	0.8
OGT	0.89	0.8
PARP1	0.92	0.8
PARP9	0.9	0.8
POMGNT1	0.93	0.8
PYGL	0.92	0.8
ST6GAL1	0.92	0.8
TIPARP	1.1	0.8
TNKS2	1.1	0.8
UGT2B7	0.89	0.8
A4GNT	0.95	0.9
ALG6	1.1	0.9
FUT4	1.1	0.9
FUT8	0.97	0.9
GCNT2	0.98	0.9
PARP2	0.96	0.9
PNP	0.98	0.9
POMT1	0.95	0.9
PYGB	0.94	0.9
QPRT	1.1	0.9
ST6GALNAC4	0.94	0.9
TYMP	1.1	0.9
ALG10	1	1
B3GNT5	1	1
B4GALNT3	1	1
B4GALT5	1	1

PARP11	0.98	1
PARP3	1	1
