# Supplementary material for: Exploration of glycosyltransferases mutation status in cervical cancer reveals PARP14 as a potential prognostic marker
Source: Glycoconj J. 2023 Aug 31;40(5):513–22. doi: 10.1007/s10719-023-10134-7 (PMC10638145; doi:10.1007/s10719-023-10134-7)
Supplement: Supplementary file 3 — Supplementary Material 3 [file 10719_2023_10134_MOESM3_ESM.docx]

**Title: Exploration of glycosyltransferases mutation status in cervical cancer reveals PARP14 as a potential prognostic marker**

Hui Wang ^1✝^, Shen Luo ^1✝^, Xin Wu ^1^, Yuanyuan Ruan ^2^, Ling Qiu ^1^, Hao Feng ^1^, Shurong Zhu ^1^, Yanan You ^1^, Ming Li ^1^, Wenting Yang ^3^, Yanding Zhao ^4^, Xiang Tao ^1^, Hua Jiang ^1,^*

1. Obstetrics & Gynecology Hospital of Fudan University, Shanghai 200090, China;
2. Department of Biochemistry and Molecular Biology, School of Basic Medical Sciences, Fudan University, Shanghai 200032, China
3. Shanghai Genenexus healthcare technology company, Shanghai 200433, China;
4. Department of Molecular and Systems Biology, The Geisel School of Medicine at Dartmouth, Lebanon, New Hampshire 03756，USA；

***** Correspondence: [jianghua@fudan.edu.cn](mailto:jianghua@fudan.edu.cn)(H.J.).

✝ These authors contributed equally to this work.

*Abstract:* This study investigates the potential role of Glycosyltransferases (GTs) in the glycosylation process and their association with malignant tumors. Specifically, the study focuses on PARP14, a member of GTs, and its potential as a target for tumors in the diagnosis and treatment of cervical cancer. To gather data, the study used somatic mutation data, gene expression data and clinical information from TCGA-CESE dataset as well as tissue samples from cervical cancer patients. Further verification was conducted through RT-qPCR and immunohistochemistry staining on cervical cancer tissues to confirm the expression of PARP14. The study utilized Kaplan-Meier for survival analysis of cervical cancer patient and found significant mutational abnormalities in GTs. The high frequency mutated gene was identified as PARP14. RT-qPCR revealed significantly higher mRNA expression of PARP14 compared to precancerous tissue. Using IHC combined with Kaplan-Meier,patients in the PARP14 high expression group had a better prognosis than the low expression group. The study identified PARP14 as a frequently mutated gene in cervical cancer and proposed its potential role in diagnosis and treatment.

*Keywords:* Glycosyltransferase (GT); Cervical Cancer; PARP14 (Poly (ADP-ribose) polymerase member 14); Mutation

#### *Declarations*

#### Funding: This research was funded by Shanghai Shenkang Hospital Development Center's Shenkang Promotion of Clinical Skills and Clinical Innovation in Municipal Hospitals ThreeYear Action Plan (2020–2023) Major Clinical Research Project (Grant No. SHDC2020CR1048B), the General Program of National Natural Science Foundation of China (Grant No.82271654), General program of Natural Science Foundation of Shanghai (Grant No. 19ZR1407000), Pilot construction project of high level universities in Shanghai (Grant No.DGF501017-06), “ZaiDing-Le”Foundation from Beijing Kanghua Foundation for the development of Traditional Chinese and Western Medicine (Grant No. KH-2020-LJJ-008).

Competing interests: The authors have no competing interests to declare that are relevant to the content of this article.

#### *Ethics approval*

#### All procedures performed in studies involving human participants were in accordance with the ethical standards of the institutional and/or national research committee and with the 1964 Helsinki Declaration and its later amendments or comparable ethical standards. The study was approved by Ethics Committee of Obstetrics & Gynecology Hospital of Fudan University (protocol code 2021-210, Nov.29, 2021 and protocol code 2022-51, Mar. 8, 2022).

*Acknowledgments*

In addition to the listed authors, we acknowledge the following individuals and teams for their assistance with this study: NHC Key Laboratory of Glycoconjugates Research, Department of Biochemistry and Molecular Biology, School of Basic Medical Sciences, Fudan University; Leading Talents Project of Huangpu District of Shanghai District Committee and Government.

1. Introduction

Glycosyltransferases (GTs) are a large family of enzymes that play an important role in the process of glycosylation. [1]. GTs are present in the endoplasmic reticulum (ER) and Golgi apparatus. Currently, more than 200 genes encoding GTs have been identified and classified into different families such as fucosyltransferase, sialyltransferase and N-acetylglucosaminyltransferase [1,2]. Previous studies have suggested that the abnormal expression of GT may be associated with various diseases, especially related with the proliferation, invasion, metastasis, epithelial-mesenchymal transition (EMT) and chemoresistance in malignant tumor[3-7]. Therefore, GTs have been suggested as crucial biomarkers for the timely detection and prediction of prognosis in various types of cancer. [4,5,7].

Cervical cancer is one of the malignant tumors of the reproductive system that seriously threatens women's health. Its incidence and mortality rate are ranked in the 5th and 4th place in the female population, and the trend is still increasing[8,9]. Recent studies have shown that the glycosylation varients are closely related to the proliferation, metastasis and drug resistance of cervical cancer [10,11] and certain GTs have been associated with diagnosis and prognosis [12]. These fingdings suggest that GTs may play a significant role in cervical cancer and could potentially become new targets for diagnosis and treatment.

Poly (ADP-ribose) polymerase member 14 (PARP14), also known as ARTD8, BAL2, or CoaST6, belongs to the group of glycosyltransferases (GTs). In the past years, PARP14 was found to be associated with STAT6 activation, B-cell differentiation, and the JNK1/JNK2 signaling pathway. There has been growing interest in PARP14 as a potential target in allergic inflammation and tumors [13].PARP14’s role in inflammatory diseases including atopic dermatitis (AD), emphysema and asthma [14-16].PARP14 also plays an important role in tumors. PARP14 high expression in human primary pancreatic cancer (PCa) is associated with poor prognosis, and PARP14 promotes the proliferation and gemcitabine chemoresistance of pancreatic cancer cells through activation of NF-κ pathway [17]. In hepatocellular carcinoma (HCC) cells, PARP14 plays a role in promoting cell survival. One way it does this is by inhibiting the activity of JNK1, which is a pro-survival protein that functions as a serine/threonine kinase. This inhibition leads to a suppression of the nuclear function of downstream PKM2, which ultimately promotes the survival of tumor cells [18].

This paper analyzed 244 GTs that were previously identified [19] and examined the mutation and expression levels of GTs in both cervical cancer patients from the Cancer Genome Atlas (TCGA) database and clinical patients. The study identified several GTs that were linked to either positive or negative prognosis. In addition, the paper delves into the role of PARP14in the prognosis of cervical cancer.

2. Materials and Methods

*TCGA dataset:* We obtained a dataset from The Cancer Genome Atlas (TCGA, [https://portal.gdc.cancer.gov)](https://portal.gdc.cancer.gov).) consisting of 178 tumor samples with somatic mutation data, 3 normal samples and 306 tumor samples with gene expression data and clinical information. The dataset was selected from TCGA-CESE. This study adheres to the publication guidelines provided by TCGA (http://cancergenome.nih.gov/publications/ publicationguidelines).

*Clinical tissue specimens:* The use of human tumor specimen was approved by the local Ethics Committee and informed consents were obtained from all patients. The clinical cohort contained data from 10 cervical cancer patients from the Obstetrics and Gynecology Hospital of Fudan University. We collected the clinical characteristic of the 10 patients of cervical cancer, with a mean age of: 51.8 years (Table 1). Of the 10 patients, 8 had squamous cell carcinoma and 2 had adenocarcinoma. Additionally, there were 5 patients in stage I, 2 in stage II and 3 in stage III (Table 1). We performed whole genome sequencing (WGS) on the samples to obtain data related to their somatic mutations. Additionally, we established a cohort of 35 cervical cancer samples and a cohort of 4 cervical cancer tissues which were matched with para-cancer tissues using immunohistochemistry staining and RT-qPCR, respectively. All clinical specimens were untreated with preoperative chemotherapy or radiotherapy.

Table 1. Patient Characteristics Associated with Cervical Cancer of Clinical Cohort for WGS

| **Characteristic** | **No. (%)** |
| --- | --- |
| Age (years), mean（range） | 51.80（35-75） |
| ≥70 | 1（10.00） |
| 50-69 | 4（40.00） |
| 35-49 | 5（50.00） |
| ＜35 | 0（0.00） |
| Body mass index（kg/m2） | 23.85（18.69-26.44） |
| BMI<18.5 | 0（0.00） |
| 18.5≤BMI＜24 | 4（40.00） |
| BMI≥24 | 6（60.00） |
| ECOG performance status score |  |
| 0 | 9（90.00） |
| 1 | 1（10.00） |
| ≥2 | 0（0.00） |
| Menstruation |  |
| Menopause | 5（50.00） |
| Pre-menopause | 5（50.00） |
| Marital status |  |
| Single | 0（0.00） |
| Married or Divorced | 10（100.00） |
| Histologic subtypes |  |
| Squamous carcinoma | 8（80.00） |
| Adenocarcinoma | 2（20.00） |
| FIGO stage |  |
| I | 5（50.00） |
| II | 2（20.00） |
| III | 3（30.00） |
| IV | 0（0.00） |

*Quantitative Real-Time PCR Analysis:* The mRNA of cervical cancer tissue was isolated and reverse transcripted to cDNAs by the MolPure® Cell/Tissue Total RNA Kit (19221ES50, Yeasen, Shanghai, China) and Hifair® Ⅱ 1st Strand cDNA Synthesis Kit (gDNA digester plus) (11123ES10, Yeasen, Shanghai, China). Then the cDNAs was used for quantitative PCR analysis using Hieff® qPCR SYBR Green Master Mix(Low Rox Plus) (11202ES03, Yeasen, Shanghai, China).The RNA concentration and quality were determined by NanoDrop 2000 (Applied Biosystems, USA)and the RT-qPCR analysis was performed on QuantStudio5 Real-Time PCR (Applied Biosystems, USA). The PCR primers were as follows: GAPDH forward, 5’-GGAGCGAGATCCCTCCAAAAT-3’ and reverse, 5’-GGCTGTTGTCATACTTCTCATGG-3’; PRAP14 forward, 5’ -TGTTAGTGGAGAACATAAGTGGC-3’ and reverse, 5’ -TGAATGGTGCTTGGTACAATCAT-3’. GAPDH was used as the internal control. Data were analyzed using the 2-ΔΔCt method.

*Immunohistochemistry (IHC) Staining:* The samples from the tissue were embedded in paraffin. Tissue sections (3μm) were prepared for IHC analyses of PARP14. In brief, the sections were de-paraffinized and, dehydrated, and were then subjected to antigen retrieval. Endogenous peroxidase was eliminated by the use of 3% H2O2.The slides were then incubated with anti-PARP14(1:100, Rabbit, Affinity, DF14173) overnight at 4℃. The samples were incubated with a second antibody (Mouse anti-rabbit IgG, Recordbio, RC0080RM) at room temperature, developed with DAB, counterstained with [hematoxylin](javascript:;) for 3min, and photographed under a microscope. All images were acquired and processed in TIFF format, and the integrated option densities (IODs) of in each image were counted and measured using ImageJ (1.53t, Java 1.8.0_322) and IHC-Toolbox plugin [^20^].

*Gene set enrichment analysis (GSEA):* Gene set enrichment analysis (GSEA) is a commenly used computational method for analyzing genome-wide expression matrices [21]. In this study, we utilized GSEA to assess related pathways and molecular mechanisms in cervical cancer patients by setting GTs gene mutation levels as population phenotypes. The nominal P-value and normalized enrichment score (NES) were used to sort the pathways enriched in each phenotype. Gene sets with a nominal P value of less than 0.05 were considered statistically significant.

*Statistics Analysis:* The R software (4.0.2, http://www.R-project.org, RRID:SCR_001905) was used to derive all statistical analyses. Somatic mutation data for 178cervical cancer samples from TCGA-CESE cohort and 10 samples from the clinical cohort were extracted by “Maftools” plugin. Overall survival (OS) was predicted using Kaplan-Meier survival plots. GraphPad Prism V8.0.1 (GraphPad Software, Inc., La Jolla, CA, USA, RRID:SCR_002865) software was used to determine statistical differences by using the Welch’s t test or one-way analysis of variance (ANOVA). p<0.05 was considered to be statistically significant.

3. Results

3.1. Glycosyltransferase (GT) Genes Associated with Mutation in TCGA Cohorts

We analyzed the association between the mutation of 244 GTs and cervical cancer in TCGA cohorts. Somatic mutation profiles from 178 cervical cancer patients were downloaded and analyzed using “Maftools” plugin. The mutations were classified into different categories with missense mutations accounting for the largest proportion based on protein structure (Figure 1A). Here we presented the top 30 mutated genes in cervical cancer, ranked by percentage, the top 10 including PARP14, ALG13, UGT2B15, ART4, C1GALNT1C1, GALNT9, MGAT4C, OGT, TNKS2, UGGT2 (Figure 1B). In addition, single nucleotide polymorphisms (SNPs) occurred more frequently than insertions or deletions (Figure 1C), with G>A being the most common single nucleotide variant (SNV) in cervical cancer (Figure 1D) according to gene structure classification.


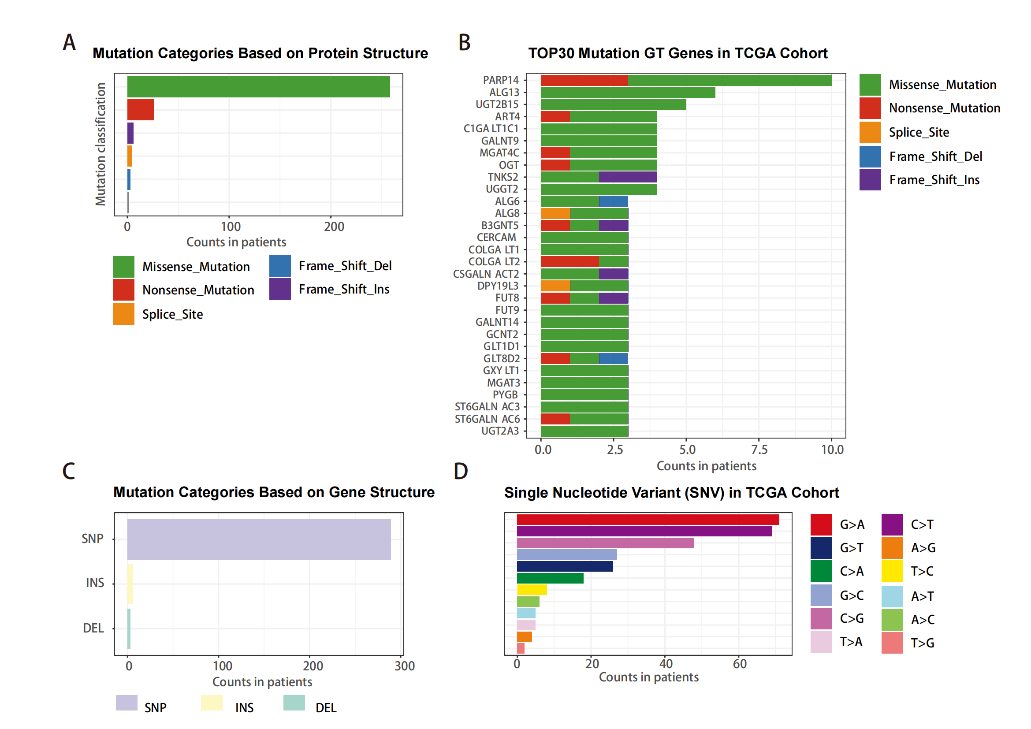


Fig. 1 Summary of the GT mutation information from TCGA (A) Mutation types were classified by protein structure categories, with missense mutations accounting for the largest proportion. (B) The top 30 mutated GT genes in cervical cancer. (C, D) Mutation types were classified by gene structure categories, SNP occurring more frequency than insertion or deletion, and G>A being the most common mutation type in SNV

3.2. Enrichment analysis of GTs based on TCGA cohort

To investigate the potential mechanism of GT effect on cervical cancer, we utilized Gene Set Enrichment Analysis (GSEA) to examine the pathways and biological processes associated with GTs. We divided the 178 cervical cancer samples from the TCGA database into mutation and non-mutation groups based on the mutation status of 244 GTs. Out of these patients, 104 were classified as the GTs mutant-type group while 90 were classified as the GTs wild-type group. As shown, we obtained significant enrichment in cell cycle (NES=1.87, P=8e-7), HPV infection (NES=2.03, P=1e-7) and T cell (NES=1.76, P=0.03) pathways (Figure 2A,B,D). In HPV infection analysis, we further found that HPV31 infection was higher in mutant-type than wild-type (Figure 2C). In our investigation of T cell-related pathways, we observed an increase in T.helper cell and M1 macrophage in mutant-type(Figure 2E), and further analysis revealed that the increase in SNV neoantigen may be the reason for the high T cell content(Figure 2F). In addition, the ratio of silent and non-silent mutations was significantly elevated in the mutant-type (Figure 2G). Finally, we identified the gene sets with the most significant differences in the enrichment of GSEA genes, including histone modifications, doxorubicin resistance, early T lymphocyte, metastasis, cell cycle, HPV positive tumors and G2M cell cycle(Figure 2H).


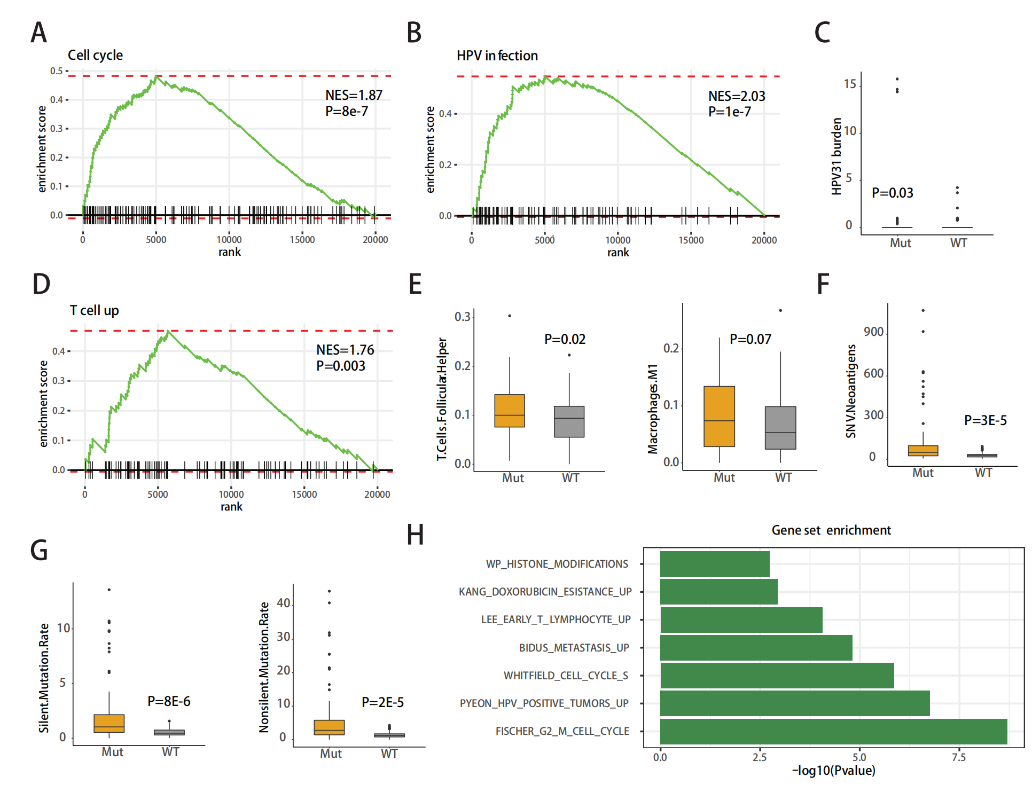


Fig. 2 Enrichment analysis of GTs in TCGA cohort (A) GSEA Enrichment plot of Cell cycle in mutation group. (B) GSEA Enrichment plot of HPV infection in mutation group. (C) Comparison of HPV31 burden between GTs mutant-type and wild-type cervical cancer patients in TCGA cohorts. (D) GSEA Enrichment plot of T cell in mutation group. (E) Comparison of T-helper cells follicular and M1 macrophages between GTs mutant-type and wild-type cervical cancer patients in TCGA cohorts. (F) SNV-induced neoantigen was significantly higher in the mutant group, which may be the reason for the increase in T cells. (G) Both silent and non-silent mutations were significantly increased in mutant-type compared to wild-type. (H) Name of the gene set with the most significant enrichment differences in the GEEA gene set.

3.3. Glycosyltransferase (GT) Genes Associated with Mutation in Clinical Cohorts

We conducted whole genome sequencing of 10 cervical cancer tissues, and obtained somatic mutation data for analysis. These mutations were classified into different categories, with the largest proportion of intron variants based on protein structure (Figure 3A). We identified the top 30 mutated genes, sorted by percentage, and the top 10 included MGAT4C, ST8SIA1, GLT1D1, B4GALNT3, GALNT13, GALNTL6, GXYLT1, ST6GALNAC3, GTDC1, XYLT (Figure 3B). In addition, SNP occurred more frequently than insertions or deletions (Figure 3C), with G>A being the most common SNV in cervical cancer (Figure 3D). To compare with the mutations in the TCGA cohort, we screened for GT genes with missense mutations. A total of 12 GT genes in the clinical cohort had missense
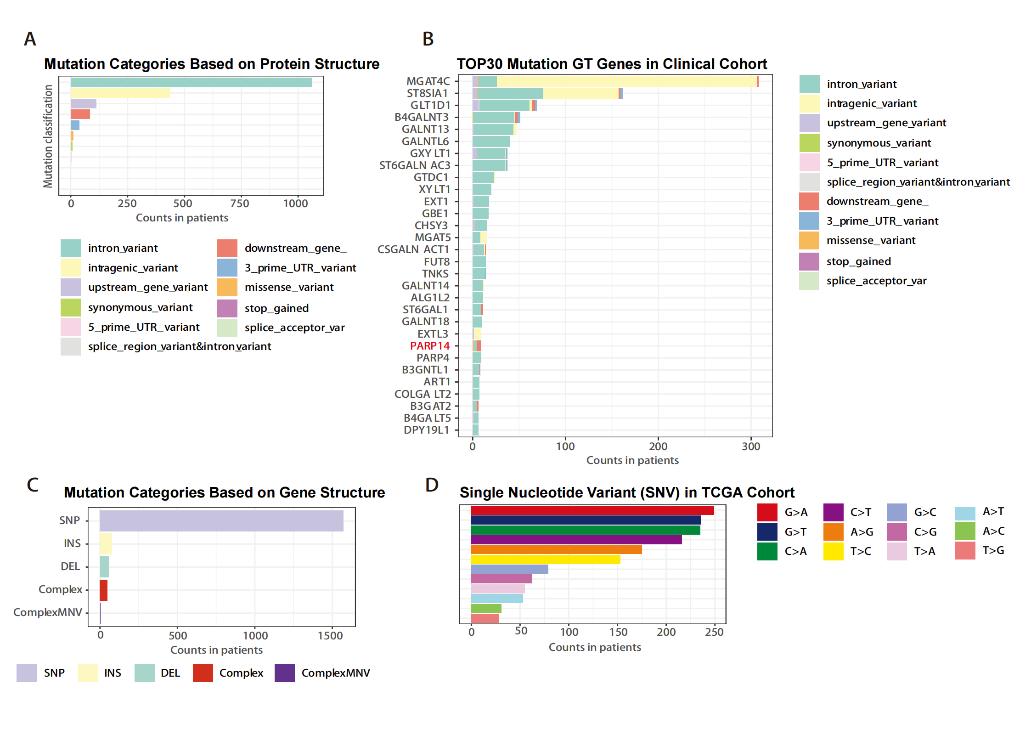
mutations, including PARP14 (Table 2).

Fig. 3 Somatic mutation patterns of GTs identified by whole-genome sequencing (WGS) in 10 patients of cervical cancer. (A) Mutation types were classified by protein structure categories, with intron variant accounting for the largest proportion. (B) The top 30 mutated GT genes in cervical cancer. (C, D) Mutation types were classified by gene structure categories, SNP occurring more frequency than insertion or deletion, and G>A being the most common mutation type in SNV.

Table 2. Glycosyltransferase Genes with Missense Mutations in Clinical Cohort For WGS.

| **Glycosyltransferase (GT) Genes** |
| --- |
| A4GNT |
| ALG10 |
| ALG10B |
| B3GALNT2 |
| DPY19L2 |
| GALNT6 |
| LFNG |
| MGAT4C |
| PARP12 |
| PARP14 |
| ST6GAL2 |
| ST8SIA1 |

3.4. Somatic mutation of GTs from patients with cervical cancer in TCGA and clinical cohort


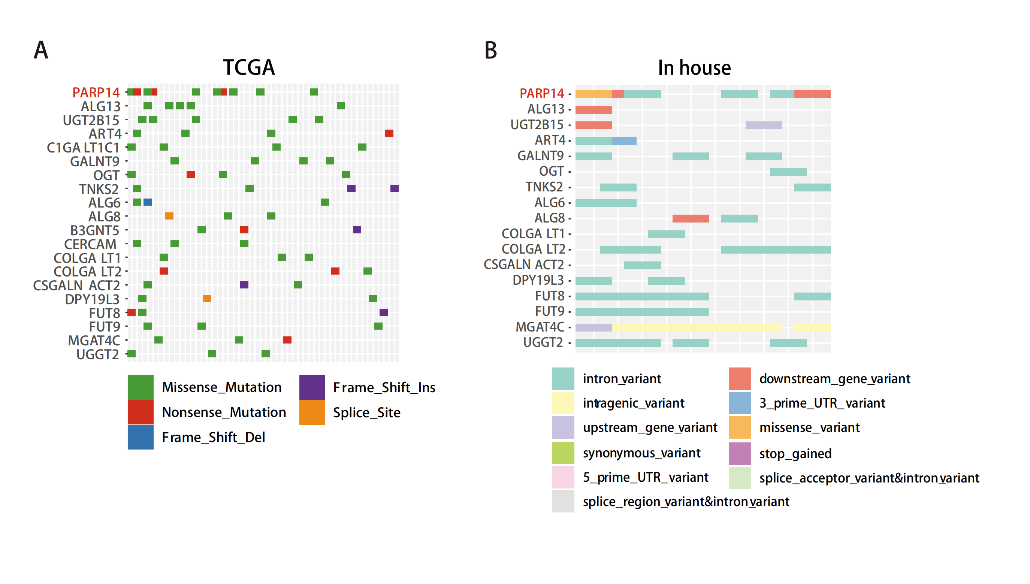
Furthermore, we summarized the mutation categories of the most frequently mutated GTs in TCGA datasets and tissues (Figure 4A, B). Notably, PARP14 had a high frequency of missense mutations in both cohorts, despite not having a higher mutation frequency in the tissue cohort. This [arouse our interest](javascript:;) in investigating the role of PARP14 in cervical cancer, leading to the following related studies.

Fig. 4 Mutation and prognosis analysis of glycosyltransferase PARP14 in cervical cancer. (A, B) The most recurrently mutated GTs genes and the somatic mutation profiles identified from TCGA and tissues of cervical cancer patients. Cases are shown in columns, genes in rows.

3.5. PARP14 expression in cervical cancer tissues and correlation analysis

According to TCGA datasets, there was an up-regulation of PARP14 in cervical cancer when compared to normal tissues (Figure 5A). And it was also validated that the mRNA expression of PARP14 in tumor tissues was significantly increased when compared to the matched para-cancer tissues (Figure 5B). To investigate the association between glycosyltransferase mutations and cervical cancer prognosis, we further conducted a survival analysis of 244 GTs in the TCGA cohort. Our analysis revealed that the majority of the 244 GTs had either a protective or hazardous effect on the prognosis of cervical cancer (Online Resource 1, Online Resource 2). We listed the 25 GT genes in which mutations had the greatest protective or hazardous effect on the prognosis of cervical cancer, with mutations in the PARP14 gene exhibiting a protective effect against cervical cancer (Figure 5C). At the same time, we performed immunohistochemical staining of cancer tissues from a clinical cohort of 35 cervical cancer patients to analyze the qualitative and quantitative expression levels of PARP14. Clinical characteristics and prognostic data were collected. The median follow-up time was 23 months. We divided them into PARP14 high expression group (N=17) and PARP14 low expression group (N=18) (Figure 5D) based on quantitative immunohistochemical analysis. The study utilized the Kaplan-Meier method to assess the prognosis of two groups, and the results were statistically different (p=0.036), with patients in the high PARP14 expression group having a better prognosis compared to those in the low PARP14 expression group (Figure 5E). In addition, we also conducted a differential analysis based on various factors such as pathological type, clinicopathological stage, menopause and body mass index (BMI). However, there was no significant difference in PARP14 expression among any of these factors(Table3).


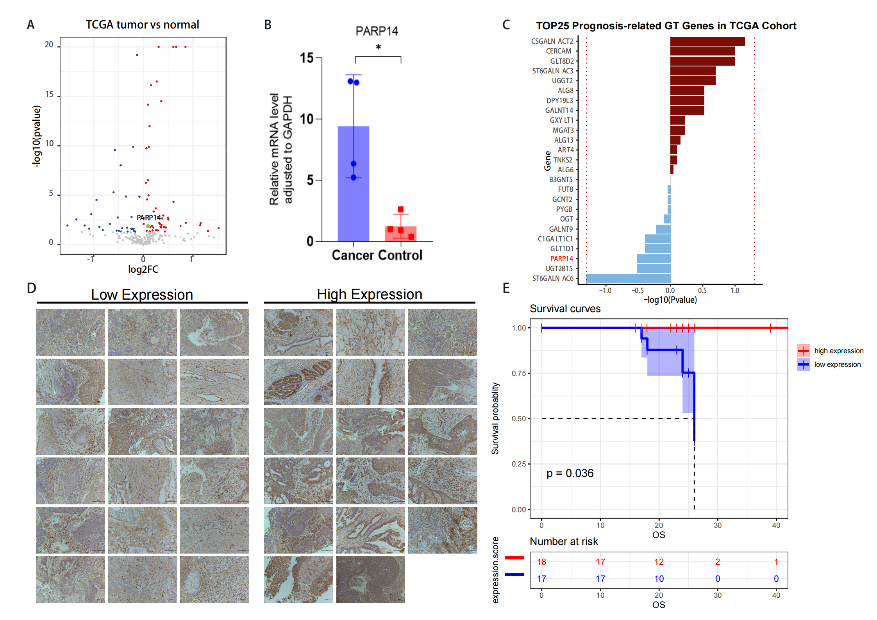


Fig. 5 PARP14 expression in cervical cancer tissues and correlation analysis. (A) Volcano plot of mRNA expression of GTs in tumor and normal cervical tissues from TCGA dataset. In the volcano map, red: genes up-regulated in tumor group; blue: genes down-regulated in tumor groups; gray: no differentially expressed genes in the tumor group; green: PARP14. (B) PARP14 expression in tumor and normal cervical tissues. (C) GT genes which had the greatest protective or hazardous effect on the prognosis of cervical cancer from TCGA cohort. (D) Immunohistochemical staining of PARP14 protein was performed on 35 cervical cancer tissues, which were divided into low expression group (N=18) and high expression group (N=17) according to expression level. (E) Kaplan-Meier survival curves for overall survival of cervical cancer patients from two groups.

Table 3. Patient Characteristics Associated With Cervical Cancer Of Clinical Cohort For IHC.

| **Characteristic** | **Total**  **(n=35)** | **High expression**  **(n=17 )** | **Low expression**  **(n=18 )** |
| --- | --- | --- | --- |
|  | **No. (%)** | **No. (%)** | **No. (%)** |
| Age (years)，mean（range） | 50.94（31-72） | 51.41（31-72） | 50.50（36-72） |
| ≥70 | 2（5.71） | 1（5.88） | 1（5.56） |
| 50-69 | 18（51.43） | 9（52.94） | 9（50.00） |
| 35-49 | 13（37.14） | 5（29.41） | 8（44.44） |
| ＜35 | 2（5.71） | 2（11.76） | 0（0.00） |
| Body mass index（kg/m2） | 24.19  （20.51-32.47） | 24.06  （20.51-32.47） | 24.33  （19.63-29.90） |
| BMI<18.5 | 0（0.00） | 0（0.00） | 0（0.00） |
| 18.5≤BMI＜24 | 22（62.86） | 11（64.71） | 11（61.11） |
| BMI≥24 | 13（37.14） | 6（35.29） | 7（38.89） |
| ECOG performance status score |  |  |  |
| 0 | 34（97.14） | 16（94.12） | 18（100.00） |
| 1 | 1（2.86） | 1（5.88） | 0（0.00） |
| ≥2 | 0（0.00） | 0（0.00） | 0（0.00） |
| Menstruation |  |  |  |
| Menopause | 14（40.00） | 6（35.29） | 8（44.44） |
| Pre-menopause | 21（6.000） | 11（64.71） | 10（55.56） |
| Marital status |  |  |  |
| Single | 2（5.71） | 1（5.88） | 1（5.56） |
| Married or divorced | 33（94.29） | 16（94.12） | 17（94.44） |
| Histologic subtypes |  |  |  |
| Squamous carcinoma | 28（80.00） | 13（76.47） | 15（83.33） |
| Adenocarcinoma | 5（14.29） | 2（11.76） | 3（16.66） |
| Adenosquamous carcinoma | 2（5.71） | 2（11.76） | 0（0.00） |
| FIGO stage |  |  |  |
| I | 12（34.29） | 6（35.29） | 6（33.33） |
| II | 10（28.57） | 3（17.65） | 7（38.89） |
| III | 13（37.14） | 8（47.06） | 3（16.66） |
| IV | 0（0.00） | 0（0.00） | 0（0.00） |
| Follow-up (month), median (range) | 23（0-42） | 23（17-26） | 23（0-42） |

*4. Discussion*

Glycosyltransferases are a large group of enzymes which play a crucial role in glycosylation. Over 200 GTs have been identified and their abnormal expression has been closely related to tumors. In colorectal cancer, the expression of GTs has been found to contribute significantly to tumor cell proliferation, survival, stem-like cell properties induction, epithelial-mesenchymal transition (EMT), metastasis and resistance to chemotherapy and radiotherapy [4]. Jahanshah Ashkani et al. analyzed that the expression of 210 GT genes from 1893 cancer patient samples in The Cancer Genome Atlas (TCGA) microarray data. Their findings showed that these genes were effective in classifying six types of cancers, including breast, ovarian, glioblastoma, kidney, colon and lung. Furthermore, these GT genes were able to classify subgroups of breast cancer [22]. Yousra Mohamed Abd-El-Halim et al. proposed a gene signature funded based on 19 GT genes that can effectively stratify the prognosis of patients with pancreatic ductal adenocarcinoma (PDAC) [23]. These studies indicates that the expression of GTs has an impact on the fate of tumors.

Our study summarized the mutation pattern of GTs in cervical cancer tissues based on TCGA database and whole genome sequencing (WGS). According to the analysis of TCGA database, it revealed that missense mutations were the most common protein structure mutation types in GTs, with the top 30 mutation frequencies all having missense mutations. Meanwhile, the number of SNPs was also more than other gene structure mutation types. Missense variants were the most common consequence of SNPs in humans [24]. We concerned that the effect of missense mutations on glycosyltransferase activity was arguably more difficult to judge than in the case of mutations with other consequences (such as total gene deletions or point nonsense mutation) [25]. Among the top 30 GTs in terms of mutation frequency, a de-novo missense mutation in C1GALT1C1: c.266 C>T, p. (T89I) caused a novel X-linked form of atypical hemolytic-uremic syndrome (aHUS) [26]. Another study by Marilena De Mariano et al. revealed that a GALNT14 mutation (c.802C > T) had been associated to the development of neuroblastoma (NB) and GALNT14 might act as a novel gene potentially involved in NB predisposition [27].

Recent studies confirmed the correlation between GTs and cervical cancer. UDP-glucose ceramide glycosyltransferase (UGCG) contributed to proliferation and glycolysis of cervical cancer cells by regulating the PI3K/AKT pathway [10]. Additionally, [Lixia Zhou](https://pubmed.ncbi.nlm.nih.gov/?term=Zhou+L&cauthor_id=36110252) et al. demonstrated an increase in the mRNA expression and protein level of GALNT2 in cervical high-grade intraepithelial neoplasia and tumor tissues compared to normal cervix tissues. GALNT2 was associated with worse overall survival and could be utilized as a prognostic biomarker for cervical cancer [12]. The findings of the current study confirmed that there was a correlation between the expression of GTs and the prognosis of cervical cancer. Out of the 244 GTs analyzed, almost all of them were found to have either a protective or detrimental effect on the prognosis of cervical cancer.

PARP14, a member of the glycosyltransferase family, has been shown to be closely associated with the development of a number of tumors, particularly in pancreatic cancer, hepatocellular carcinoma, and hematologic malignancy [17,18,28-30]. In diffuse large B-cell lymphoma (DLBCL), PARP14 regulated the IL-4-STAT6 signaling pathway, enhanced the expression of several downstream genes, and enabled DLBCL tumor cell survival [31]. In this study, we found that PARP14 showed a trend of high expression in cervical cancer tissues compared to para cancerous tissues, and the high expression of PARP14 was closely related to the prognosis of patients, suggesting that patients with high PARP14 expression have a better prognosis than those with low expression. Although our case sample was limited and our study was restricted to a descriptive study, the result still led us to focus on the role of PARP14 in cervical cancer. GSEA enrichment analysis identified cell cycle, HPV infection, and T cell-associated immunity as the probable mechanistic pathways. It had been demonstrated that cyclin D1 was over-expressed in many tumors. PARP14 deletion led to a decrease of cyclin D1, resulting in G1 cell-cycle arrest and reduced proliferation [32]. This confirmed the involvement of PARP14 in regulating cell cycle progression. However, the role of this mechanism in cervical cancer was unclear. Among many allergic diseases, PARP14 interacted with STAT6 to enhance IL-4-induced gene expression in T cells, thereby promoting Th2 differentiation [33,34]. Research on the mechanism of PARP14 and HPV infection has not known yet. Regrettably, our study has not yet established a correlation between mutation and expression of PARP14 in cervical cancer. This would hold significant importance for future studies. The potential mechanism of PARP14 in cervical cancer will be explored in our further studies.

*5. Conclusions*

In this study, we analyzed the high-frequency mutated GT gene PARP14 from both TCGA database and clinical case samples. We found that PARP14 was significantly overexpressed at both the molecular and protein levels in cervical cancer tissues, and that higher expression of PARP14 caused better prognosis. Our findings suggested a potential role of PARP14 in the diagnosis and treatment of cervical cancer.

***Acknowledgments:*** In addition to the listed authors, we acknowledge the following individuals and teams for their assistance with this study: NHC Key Laboratory of Glycoconjugates Research, Department of Biochemistry and Molecular Biology, School of Basic Medical Sciences, Fudan University; Leading Talents Project of Huangpu District of Shanghai District Committee and Government.

***Author Contributions:*** Conceptualization, X.W. and Y.R.; methodology, H.W., S.L. and X.T.; software, H.W. and W.Y.; validation, H.F., L.Q. and S.Z.; formal analysis, Y.Z.; investigation, Y.Y.; resources, M.L.; data curation, S.L.; writing—original draft preparation, H.W.; writing—review and editing, X.W., Y.R. and H.J.; visualization, Y.Z.; supervision, H.J.; project administration, X.W. and H.J.; funding acquisition, H.J and X.W.. All authors have read and agreed to the published version of the manuscript.

***Funding:*** This research was funded by Shanghai Shenkang Hospital Development Center's Shenkang Promotion of Clinical Skills and Clinical Innovation in Municipal Hospitals Three Year Action Plan (2020–2023) Major Clinical Research Project (Grant No. SHDC2020CR1048B), the General Program of National Natural Science Foundation of China (Grant No.82271654), General program of Natural Science Foundation of Shanghai (Grant No. 19ZR1407000), Pilot construction project of high level universities in Shanghai (Grant No.DGF501017-06), “ZaiDing-Le” Foundation from Beijing Kanghua Foundation for the development of Traditional Chinese and Western Medicine (Grant No. KH-2020-LJJ-008).

***Data availability:*** The datasets generated during and/or analyzed during the current study are available from the corresponding author on reasonable request.

**Compliance with ethical standards**

***Conflicts of Interest:*** The authors declare no conflict of interest. The funders had no role in the design of the study; in the collection, analyses, or interpretation of data; in the writing of the manuscript; or in the decision to publish the results.

#### *Ethics approval:* All procedures performed in studies involving human participants were in accordance with the ethical standards of the institutional and/or national research committee and with the 1964 Helsinki Declaration and its later amendments or comparable ethical standards. The study was approved by Ethics Committee of Obstetrics & Gynecology Hospital of Fudan University (protocol code 2021-210, Nov.29, 2021 and protocol code 2022-51, Mar. 8, 2022).

*References*

1. In: Varki A, Cummings RD, Esko JD, et al, eds. *Essentials of Glycobiology*. 4th ed. 2022.

2. Taniguchi N, Kizuka Y. Glycans and cancer: role of N-glycans in cancer biomarker, progression and metastasis, and therapeutics. *Adv Cancer Res*. 2015;126:11-51. doi:10.1016/bs.acr.2014.11.001

3. Ohtsubo K, Marth JD. Glycosylation in cellular mechanisms of health and disease. *Cell*. Sep 8 2006;126(5):855-67. doi:10.1016/j.cell.2006.08.019

4. Fernandez-Ponce C, Geribaldi-Doldan N, Sanchez-Gomar I, et al. The Role of Glycosyltransferases in Colorectal Cancer. *Int J Mol Sci*. May 30 2021;22(11)doi:10.3390/ijms22115822

5. Fernandez LP, Sanchez-Martinez R, Vargas T, et al. The role of glycosyltransferase enzyme GCNT3 in colon and ovarian cancer prognosis and chemoresistance. *Sci Rep*. May 31 2018;8(1):8485. doi:10.1038/s41598-018-26468-4

6. Li N, Xu H, Fan K, et al. Altered beta1,6-GlcNAc branched N-glycans impair TGF-beta-mediated epithelial-to-mesenchymal transition through Smad signalling pathway in human lung cancer. *J Cell Mol Med*. Oct 2014;18(10):1975-91. doi:10.1111/jcmm.12331

7. Shan M, Yang D, Dou H, Zhang L. Fucosylation in cancer biology and its clinical applications. *Prog Mol Biol Transl Sci*. 2019;162:93-119. doi:10.1016/bs.pmbts.2019.01.002

8. Sung H, Ferlay J, Siegel RL, et al. Global Cancer Statistics 2020: GLOBOCAN Estimates of Incidence and Mortality Worldwide for 36 Cancers in 185 Countries. *CA Cancer J Clin*. May 2021;71(3):209-249. doi:10.3322/caac.21660

9. Bray F, Ferlay J, Soerjomataram I, Siegel RL, Torre LA, Jemal A. Global cancer statistics 2018: GLOBOCAN estimates of incidence and mortality worldwide for 36 cancers in 185 countries. *CA Cancer J Clin*. Nov 2018;68(6):394-424. doi:10.3322/caac.21492

10. Zhang F, Zhang H. UDP-Glucose Ceramide Glycosyltransferase Contributes to the Proliferation and Glycolysis of Cervical Cancer Cells by Regulating the PI3K/AKT Pathway. *Ann Clin Lab Sci*. Sep 2021;51(5):663-669.

11. You X, Wang Y, Meng J, et al. Exosomal miR663b exposed to TGFbeta1 promotes cervical cancer metastasis and epithelialmesenchymal transition by targeting MGAT3. *Oncol Rep*. Apr 2021;45(4)doi:10.3892/or.2021.7963

12. Zhou L, Wu H, Bai X, Min S, Zhang J, Li C. O-Glycosylating Enzyme GALNT2 Predicts Worse Prognosis in Cervical Cancer. *Pathol Oncol Res*. 2022;28:1610554. doi:10.3389/pore.2022.1610554

13. Tauber AL, Levonis SM, Schweiker SS. Recent developments in PARP14 research. *Future Med Chem*. Sep 2020;12(18):1657-1667. doi:10.4155/fmc-2020-0166

14. Krishnamurthy P, Da-Silva-Arnold S, Turner MJ, Travers JB, Kaplan MH. Poly-ADP ribose polymerase-14 limits severity of allergic skin disease. *Immunology*. Nov 2017;152(3):451-461. doi:10.1111/imm.12782

15. Zaffini R, Gotte G, Menegazzi M. Asthma and poly(ADP-ribose) polymerase inhibition: a new therapeutic approach. *Drug Des Devel Ther*. 2018;12:281-293. doi:10.2147/DDDT.S150846

16. Hu WP, Zeng YY, Zuo YH, Zhang J. Identification of novel candidate genes involved in the progression of emphysema by bioinformatic methods. *Int J Chron Obstruct Pulmon Dis*. 2018;13:3733-3747. doi:10.2147/COPD.S183100

17. Yao N, Chen Q, Shi W, Tang L, Fu Y. PARP14 promotes the proliferation and gemcitabine chemoresistance of pancreatic cancer cells through activation of NF-kappaB pathway. *Mol Carcinog*. Jul 2019;58(7):1291-1302. doi:10.1002/mc.23011

18. Iansante V, Choy PM, Fung SW, et al. PARP14 promotes the Warburg effect in hepatocellular carcinoma by inhibiting JNK1-dependent PKM2 phosphorylation and activation. *Nat Commun*. Aug 10 2015;6:7882. doi:10.1038/ncomms8882

19. Joud M, Moller M, Olsson ML. Identification of human glycosyltransferase genes expressed in erythroid cells predicts potential carbohydrate blood group loci. *Sci Rep*. Apr 16 2018;8(1):6040. doi:10.1038/s41598-018-24445-5

20. Shu J, Dolman GE, Duan J, Qiu G, Ilyas M. Statistical colour models: an automated digital image analysis method for quantification of histological biomarkers. *Biomed Eng Online*. Apr 27 2016;15:46. doi:10.1186/s12938-016-0161-6

21. Subramanian A, Tamayo P, Mootha VK, et al. Gene set enrichment analysis: a knowledge-based approach for interpreting genome-wide expression profiles. *Proc Natl Acad Sci U S A*. Oct 25 2005;102(43):15545-50. doi:10.1073/pnas.0506580102

22. Ashkani J, Naidoo KJ. Glycosyltransferase Gene Expression Profiles Classify Cancer Types and Propose Prognostic Subtypes. *Sci Rep*. May 20 2016;6:26451. doi:10.1038/srep26451

23. Mohamed Abd-El-Halim Y, El Kaoutari A, Silvy F, et al. A glycosyltransferase gene signature to detect pancreatic ductal adenocarcinoma patients with poor prognosis. *EBioMedicine*. Sep 2021;71:103541. doi:10.1016/j.ebiom.2021.103541

24. Lek M, Karczewski KJ, Minikel EV, et al. Analysis of protein-coding genetic variation in 60,706 humans. *Nature*. Aug 18 2016;536(7616):285-91. doi:10.1038/nature19057

25. Cao X, Durairaj P, Yang F, Bureik M. A comprehensive overview of common polymorphic variants that cause missense mutations in human CYPs and UGTs. *Biomed Pharmacother*. Mar 2019;111:983-992. doi:10.1016/j.biopha.2019.01.024

26. Hadar N, Schreiber R, Eskin-Schwartz M, et al. X-linked C1GALT1C1 mutation causes atypical hemolytic uremic syndrome. *Eur J Hum Genet*. Jan 4 2023;doi:10.1038/s41431-022-01278-5

27. De Mariano M, Gallesio R, Chierici M, et al. Identification of GALNT14 as a novel neuroblastoma predisposition gene. *Oncotarget*. Sep 22 2015;6(28):26335-46. doi:10.18632/oncotarget.4501

28. Camicia R, Bachmann SB, Winkler HC, et al. BAL1/ARTD9 represses the anti-proliferative and pro-apoptotic IFNgamma-STAT1-IRF1-p53 axis in diffuse large B-cell lymphoma. *J Cell Sci*. May 1 2013;126(Pt 9):1969-80. doi:10.1242/jcs.118174

29. Goenka S, Cho SH, Boothby M. Collaborator of Stat6 (CoaSt6)-associated poly(ADP-ribose) polymerase activity modulates Stat6-dependent gene transcription. *J Biol Chem*. Jun 29 2007;282(26):18732-9. doi:10.1074/jbc.M611283200

30. Barbarulo A, Iansante V, Chaidos A, et al. Poly(ADP-ribose) polymerase family member 14 (PARP14) is a novel effector of the JNK2-dependent pro-survival signal in multiple myeloma. *Oncogene*. Sep 5 2013;32(36):4231-42. doi:10.1038/onc.2012.448

31. Cho SH, Goenka S, Henttinen T, et al. PARP-14, a member of the B aggressive lymphoma family, transduces survival signals in primary B cells. *Blood*. Mar 12 2009;113(11):2416-25. doi:10.1182/blood-2008-03-144121

32. O'Connor MJ, Thakar T, Nicolae CM, Moldovan GL. PARP14 regulates cyclin D1 expression to promote cell-cycle progression. *Oncogene*. Jul 2021;40(30):4872-4883. doi:10.1038/s41388-021-01881-8

33. Mehrotra P, Hollenbeck A, Riley JP, et al. Poly (ADP-ribose) polymerase 14 and its enzyme activity regulates T(H)2 differentiation and allergic airway disease. *J Allergy Clin Immunol*. Feb 2013;131(2):521-31 e1-12. doi:10.1016/j.jaci.2012.06.015

34. Mehrotra P, Riley JP, Patel R, Li F, Voss L, Goenka S. PARP-14 functions as a transcriptional switch for Stat6-dependent gene activation. *J Biol Chem*. Jan 21 2011;286(3):1767-76. doi:10.1074/jbc.M110.157768
